# Clinical features of varicella-zoster virus meningitis diagnosed by polymerase chain reaction without abnormal routine cerebrospinal fluid analysis

**DOI:** 10.1007/s13365-025-01277-4

**Published:** 2025-11-10

**Authors:** Akihiro Kitamura, Ryutaro Nakamura, Seiji Sugiyama, Ryota Tamura, Takahito Tsukamoto, Hiroyuki Yabata, Shuhei Kobashi, Yoshitaka Tamaki, Nobuhiro Ogawa, Isamu Yamakawa, Tomoya Terashima, Makoto Urushitani

**Affiliations:** 1 Department of Neurology, Clinical Neuroscience Research Unit, Molecular Neuroscience Research Center, Shiga University of Medical Science, Otsu, 520-2192 Japan; 2 Department of Neurology, Shiga University of Medical Science, Otsu, Japan

**Keywords:** Varicella-zoster virus meningitis, Normal routine cerebrospinal fluid testing, Polymerase chain reaction, The FilmArray^®^ meningitis/encephalitis panel

## Abstract

This study aimed to investigate the clinical features of varicella-zoster virus (VZV) meningitis cases diagnosed by polymerase chain reaction (PCR) despite normal routine cerebrospinal fluid (CSF) testing. A retrospective review was performed on hospitalized central nervous system (CNS) infection cases at our institution from 2013 to 2024. VZV meningitis cases were analyzed and divided into two groups: those diagnosed by positive PCR without routine CSF abnormalities (PCR Group) and those with routine CSF abnormalities (Usual Group). PCR methods included conventional techniques and a rapid detection system (FilmArray^®^). Among 75 CNS infection cases (49 viral, 13 bacterial, 6 tuberculosis, 2 fungal, and 1 amoebic), 29 VZV meningitis cases were identified. Compared to the Usual Group (*n* = 25), the PCR Group (*n* = 4) had significantly lower CSF cell counts (median 1.0 vs. 99.0/µl, *p* < 0.001) and protein levels (44.0 vs. 70.0 mg/dl, *p* < 0.001) but similar glucose levels (58.0 vs. 54.0 mg/dl, *p* = 0.17). All PCR Group cases were female (vs. 52% in the Usual Group), had a trigeminal skin rash (vs. 52%), and presented with headache without meningeal irritation signs (vs. 44.0%) or fever. 50% of cases in the PCR Group were immunocompromised (vs.24%). Other clinical and epidemiological features were similar in both groups. Routine CSF analysis may fail to reveal abnormalities in VZV meningitis, particularly in both immunocompromised and immunocompetent female patients presenting with trigeminal skin rash and headache in the absence of meningeal irritation signs or fever. PCR is recommended to facilitate prompt and accurate diagnosis in such cases.

## Introduction

Varicella-zoster virus (VZV) meningitis accounts for 0.4–13% of cases of viral meningitis (Arruti et al. 2017; Becerra et al. 2013; Grahn and Studahl 2015; Hausfater et al. 2004; Kaewpoowat et al. 2016; Kupila et al. 2006; Mørch et al. 2003), which has been reported to occur secondary to herpes zoster in approximately 0.5% of affected individuals (Kim et al. 2017). Early diagnosis and treatment are important since VZV meningitis can lead to severe complications, especially in immunocompromised patients.

Cerebrospinal fluid (CSF) analysis is essential for diagnosing VZV meningitis. Typical findings include pleocytosis with lymphocytic predominance, normal to mildly elevated protein, and normal glucose. However, in certain cases, no abnormalities may be detected in routine CSF testing (Sabbagh et al. 2022). The detection of VZV DNA in CSF, exhibiting a sensitivity of 80–95% and a specificity exceeding 95%, serves as a definitive diagnostic modality (Tunkel et al. 2008). The FilmArray^®^ Meningitis/Encephalitis Panel (ME Panel) (bioMérieux Japan Ltd.) is a valuable diagnostic tool that enables rapid polymerase chain reaction (PCR) analysis of CSF within one hour (Leber et al. 2016), and has been approved in clinical practice since September 2022 in Japan.

The absence of pleocytosis has been described with several viruses, but not well described in VZV. This study aimed to investigate the clinical features of VZV meningitis cases diagnosed by PCR despite normal routine CSF testing.

## Methods

This is a retrospective clinical-epidemiological descriptive study that included all patients admitted to our institution with a diagnosis of central nervous system (CNS) infection from 2013 to 2024. Among these, VZV meningitis cases were investigated. Patients older than 16 years were diagnosed with VZV meningitis based on the clinical features of meningitis, the presence of herpes zoster, CSF routine testing (leukocyte count, protein content, glucose content), VZV antibodies, or a VZV PCR in the CSF. Since April 2023, the Film Array^®^ ME Panel has been utilized for CSF PCR testing. We divided VZV meningitis cases into two groups: those diagnosed by positive PCR without routine CSF abnormalities (PCR Group) and those with routine CSF abnormalities (Usual Group). Normal routine CSF findings were defined as follows: leukocyte count ≤ 5 cells/ul, protein concentration within the range of 15–55 mg/dl, and a normal glucose level ≥ 50% of the corresponding serum glucose level. Medical records were reviewed, and epidemiological data, clinical manifestations, laboratory data, treatment, and outcomes were collected. We compared these two groups across these parameters. Immunocompromised status was defined as the presence of any of the following: active chemotherapy or radiotherapy for cancer, long-term use of corticosteroids or other immunosuppressive agents, HIV infection, or post-transplant immunosuppression.

All statistical analyses were conducted using EZR version 1.53 (Saitama Medical Center, Jichi Medical University, Japan). Data are presented as median [min-max]. The t-test was used to compare data between the two groups.

This research was approved by the ethics committee of Shiga University of Medical Science (No. R2025-032). Participants were given the opportunity to opt out of the study through publicly posted information.

## Results

During the study period, 75 cases of CNS infections were identified. Of these, 49 cases (65%) were viral, 13 (17%) were bacterial, 6 (8.0%) were tuberculous, 2 (2.7%) were fungal, 1 (1.3%) was amoebic, and 4 (5.4%) had undetermined etiology.

**Fig. 1:**
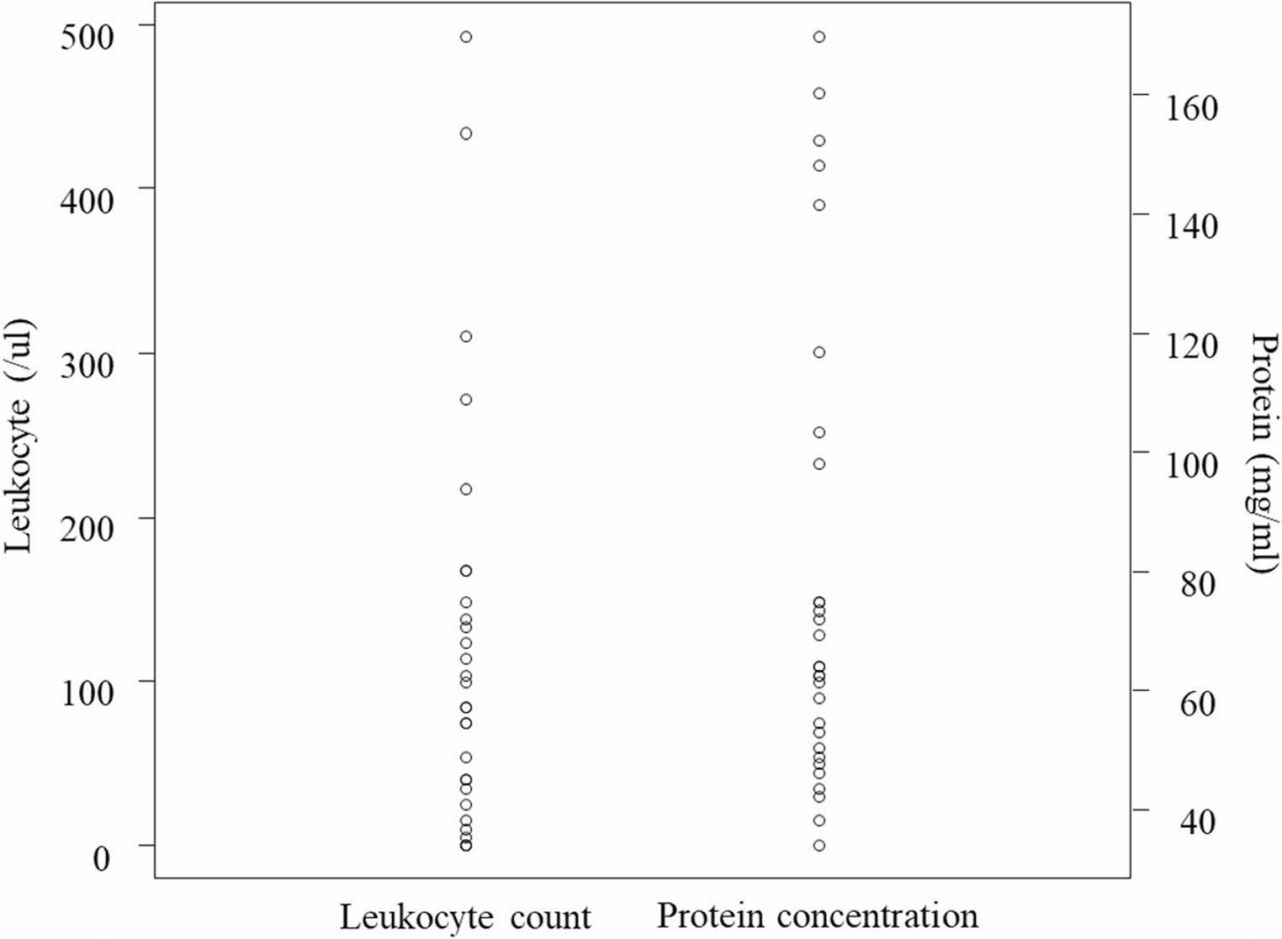
Leukocyte count and protein concentration in cerebrospinal fluid in cases with VZV meningitis

A total of 29 cases of VZV meningitis were detected. The Table 1 shows the clinical and epidemiological features of the patients. The median age was 59 years (range 16–85 years). Twelve cases (41.3%) were male and 8 cases (27.6%) were immunocompromised. The median time from symptom onset to CSF sample collection was 4 days (range 2–20). The median body temperature was 37.2℃ (range 36.0–39.0℃), and 12 cases (41.3%) had a fever above 37.5℃. Skin rash was observed in 26 cases (89.6%), of whom 17 cases (58.6%) presented rash in the trigeminal regions. Twenty-three cases (79.3%) presented with a headache, among whom 11 cases (37.9%) exhibited meningeal irritation signs. Routine CSF analysis demonstrated pleocytosis (median 85/ul, range 0-493) with lymphocytic predominance (median 99%, range 0-100%), normal to mildly elevated protein (median 64 mg/dl, range 34–170), and normal glucose (median 57 mg/dl, range 30–90) (Figure 1). All patients were hospitalized and received intravenous acyclovir therapy in accordance with Japanese clinical treatment guidelines.

**Table 1:**
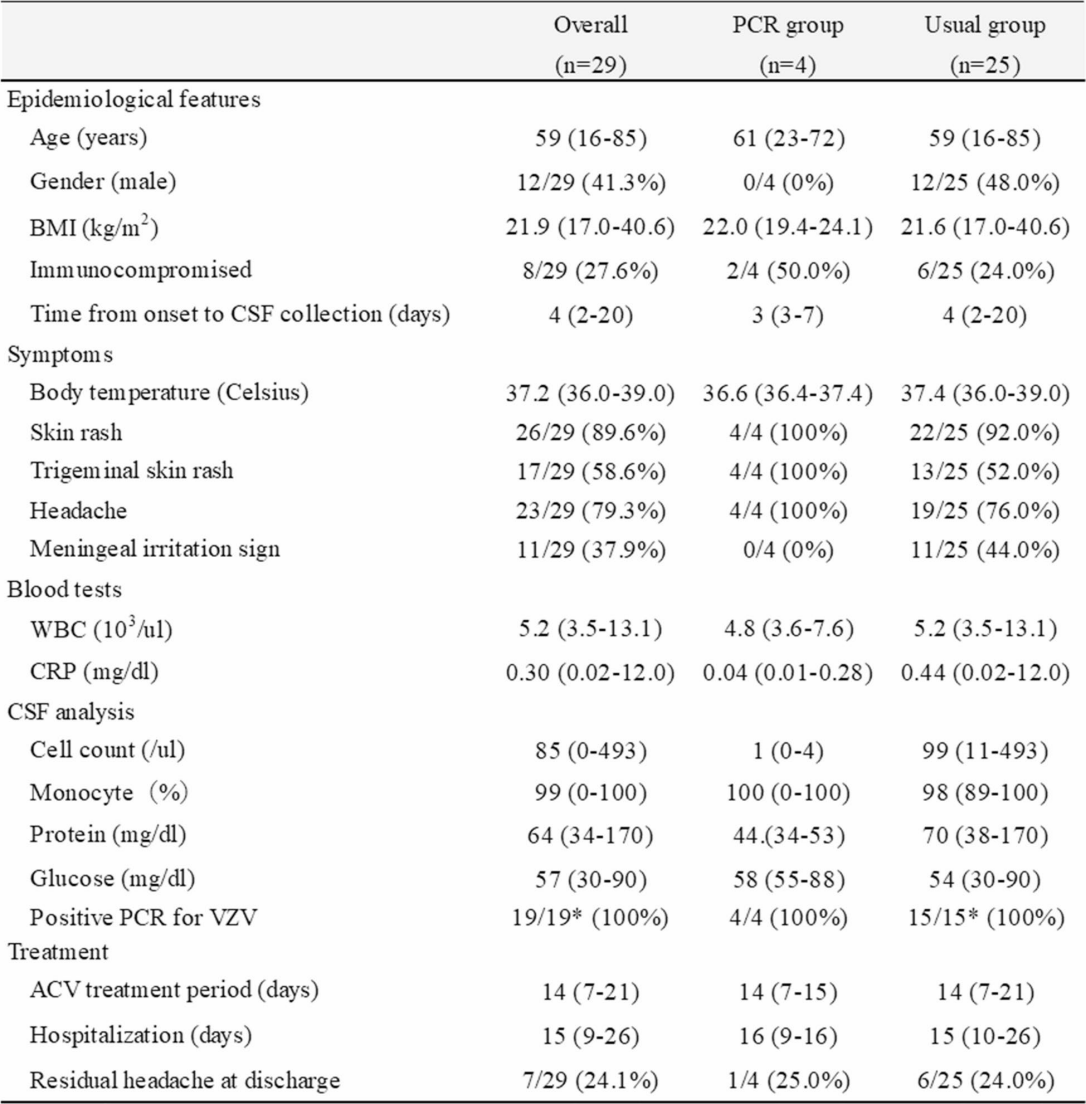
Clinical and epidemiological features of VZV meningitis patients with or without abnormal routine CSF findings

The data was shown by median [median (min-max)] or percentage (%)

PCR was conducted in 19 cases and was positive in all cases. Among these, 4 cases (13.7%) exhibited no abnormalities in routine CSF analysis (PCR Group). These were compared with the remaining 25 cases showing abnormal routine CSF findings (Usual Group). All cases in the PCR group were female (vs. 48% male in the Usual group) and presented with headache (vs. 76%) and skin rash (vs. 92%) in the trigeminal region (vs. 52%), but lacked fever or signs of meningeal irritation (vs. 44.0%). Routine CSF analysis in the PCR Group showed significantly lower cell counts (/µl) (median 1 [range 0–4] vs. 99 [11–493], *p* < 0.001) and protein concentrations (mg/dl) (44 [34–53] vs. 70 [38–170], *p* < 0.001), while glucose levels (mg/dl) were not significantly different (58 [55–88] vs. 54 [30–90], *p* = 0.17). In the PCR group, 2 out of 4 cases (50.0%) had underlying diseases associated with immunosuppression or were receiving immunosuppressive therapy, while 2 cases had no identifiable immune dysfunction (compared to 24.0% in the Usual group).

Age, body mass index (BMI), interval from symptom onset to CSF collection, inflammatory markers in blood tests, duration of acyclovir administration, length of hospitalization, and the presence of residual headache at discharge were comparable between the two groups.

## Discussion

VZV is an alpha-herpesvirus that usually causes mild to moderate presentation with disseminated yvesicular rash in primary infection. After reactivation, VZV can cause a wide range of neurologic diseases, with herpes zoster and post-herpetic neuralgia being the most frequent. Meningitis is a more severe manifestation of VZV reactivation, being more prominent in immunocompromised states and older patients. Patients mostly present with headaches, fever, lethargy, and sometimes rash that usually appears several days after local pain (Ihekwaba et al. 2008). In the present study, 29 cases of VZV meningitis were detected over an 11-year period at our institution, of which 4 (13.8%) exhibited no abnormalities in routine CSF analysis despite positive VZV PCR. All four cases, two of whom were immunocompromised, were female and presented with headache accompanied by trigeminal skin rash, but without fever or meningeal irritation signs. Notably, this occurred in both immunocompromised (*n* = 2) and immunocompetent (*n* = 2) cases.

In a review of meningitis cases without CSF pleocytosis, the most frequently identified pathogens were bacterial (99 of 124 cases), followed by viral (26 of 124 cases), and fungal organisms (9 of 124 cases) (Troendle and Pettigrew 2019). The absence of pleocytosis has been described with several viruses, but is not well described in VZV. VZV meningitis is by no means typical aseptic meningitis in which the CSF leukocyte count is not easily elevated. CSF leukocyte count in VZV meningitis has been reported to average approximately 300/ul, generally lower than that observed in herpes simplex virus meningitis but higher than in enteroviral meningitis (Ihekwaba et al. 2008; Lee et al. 2021). Several mechanisms may explain the absence of routine CSF abnormalities in our PCR-positive cases. First, immunosuppression due to underlying medical conditions or therapeutic interventions could attenuate the immune response within the CSF. However, half of our PCR group cases were immunocompetent, indicating that normal CSF can occur regardless of immune status. Second, factors previously implicated in the absence of CSF leukocytosis in pediatric enteroviral meningitis, such as older age, a short interval between symptom onset and lumbar puncture, and low peripheral leukocyte counts, showed no significant differences between the two groups in our study. Third, disease severity does not appear to be a factor, as both groups had similar outcomes, including duration of acyclovir treatment, hospitalization length, and residual symptoms such as headache. Finally, VZV reactivation with localized inflammation could be considered. Viral reactivation predominantly affects ganglia and peripheral nerves, possibly leading to minimal extension to meninges, and resulting in localized inflammation, without triggering significant CSF changes.

The clinical implications of our findings are significant. Traditional diagnostic approaches relying solely on routine CSF analysis may miss VZV meningitis cases. It should be noted that asymptomatic VZV meningitis, characterized by positive CSF findings in the absence of clinical symptoms, has been reported in 40–50% of patients with herpes zoster (Gilden et al. 2000; Nagel and Gilden 2014). In such cases, CSF analysis is not typically pursued, which can result in underdiagnosis, and consequently, intravenous acyclovir therapy may be excluded as a therapeutic option. Of course, the optimal validity of therapeutic intervention for asymptomatic VZV meningitis and for VZV meningitis identified solely through PCR positivity in the absence of routine CSF abnormalities remains insufficient and awaits further accumulation of clinical evidence.

In the evaluation of suspected meningitis in patients with antecedent herpes zoster, CSF analysis should ideally include VZV-DNA testing within 7 to 10 days of rash onset. Afterward, concurrent assessment of both VZV-DNA and VZV-IgG is advisable. VZV-DNA exhibits a sensitivity of 80–95% and a specificity exceeding 95%; however, its detectability persists for only 1 to 3 weeks following symptom onset, after which levels may decline, increasing the risk of false-negative results (Tunkel et al. 2008). VZV-specific IgG and IgM antibodies typically begin to rise 1 to 2 weeks after disease onset and can remain elevated for several months. VZV-IgG demonstrates particularly high sensitivity (approximately 93%) and is well-suited for screening purposes, serving as a valuable adjunct to mitigate potential false-negative PCR outcomes (Gregoire et al. 2006). However, PCR results are not available in time to inform decisions about using acyclovir for VZV meningitis. Recent tools for comprehensive molecular diagnosis, such as the Film Array^®^ ME Panel, are highly beneficial for accurate and timely identification (Hara et al. 2022; Tansarli and Chapin 2020; Trujillo-Gómez et al. 2022).

This study has several limitations. First, the sample size, particularly of PCR-positive patients with normal CSF findings, is small (*n* = 4), necessitating further case accumulation to validate these exploratory findings. Second, some patients with viral meningitis who tested PCR-negative may have had false-negative results due to delayed timing of CSF collection relative to symptom onset. Third, PCR testing was not performed in all VZV meningitis cases, potentially introducing selection bias. Fourth, the diagnostic methodology evolved during the study period, with the FilmArray^®^ ME Panel only becoming available in April 2023. Finally, as a single-center retrospective study, our findings may be subject to incomplete data collection and may not be generalizable to other populations or healthcare settings.,

## Conclusion

Routine CSF analysis may fail to reveal abnormalities in VZV meningitis, particularly in both immunocompromised and immunocompetent female patients presenting with trigeminal skin rash and headache in the absence of meningeal irritation signs or fever. PCR is recommended to facilitate a prompt and accurate diagnosis in such clinical scenarios.

## Data Availability

No datasets were generated or analysed during the current study.

## References

[CR1] Arruti M, Piñeiro LD, Salicio Y, Cilla G, Goenaga MA, López de Munain A (2017) Incidence of varicella Zoster virus infections of the central nervous system in the elderly: a large tertiary hospital-based series (2007–2014). J Neurovirol 23:451–459 28224485 10.1007/s13365-017-0519-y

[CR2] Becerra JC, Sieber R, Martinetti G, Costa ST, Meylan P, Bernasconi E (2013) Infection of the central nervous system caused by varicella Zoster virus reactivation: a retrospective case series study. Int J Infect Dis 17:e529–e534 23566589 10.1016/j.ijid.2013.01.031

[CR3] Gilden DH, Kleinschmidt-DeMasters BK, LaGuardia JJ, Mahalingam R, Cohrs RJ (2000) Neurologic complications of the reactivation of varicella-zoster virus. N Engl J Med 342:635–645 10699164 10.1056/NEJM200003023420906

[CR4] Grahn A, Studahl M (2015) Varicella-zoster virus infections of the central nervous system – prognosis, diagnostics and treatment. J Infect 71:281–293 26073188 10.1016/j.jinf.2015.06.004

[CR5] Gregoire SM, van Pesch V, Goffette S, Peeters A, Sindic CJ (2006) Polymerase chain reaction analysis and oligoclonal antibody in the cerebrospinal fluid from 34 patients with varicella-zoster virus infection of the nervous system. J Neurol Neurosurg Psychiatry 77:938–942 16844949 10.1136/jnnp.2006.090316 PMC2077607

[CR6] Hara M, Ishihara M, Nakajima H (2022) Use of the FilmArray^®^ meningitis/encephalitis panel to detect pathogenic microorganisms in cerebrospinal fluid specimens: a single-center retrospective study. J Int Med Res 50:3000605221129561 36214109 10.1177/03000605221129561 PMC9551344

[CR7] Hausfater P, Fillet AM, Rozenberg F, Arthaud M, Trystram D, Huraux JM, Lebon P, Riou B (2004) Prevalence of viral infection markers by polymerase chain reaction amplification and interferon-alpha measurements among patients undergoing lumbar puncture in an emergency department. J Med Virol 73:137–146 15042661 10.1002/jmv.20068

[CR8] Ihekwaba UK, Kudesia G, McKendrick MW (2008) Clinical features of viral meningitis in adults: significant differences in cerebrospinal fluid findings among herpes simplex virus, varicella Zoster virus, and enterovirus infections. Clin Infect Dis 47:783–789 18680414 10.1086/591129

[CR9] Kaewpoowat Q, Salazar L, Aguilera E, Wootton SH, Hasbun R (2016) Herpes simplex and varicella zoster CNS infections: clinical presentations, treatments and outcomes. Infection 44:337–345 26680781 10.1007/s15010-015-0867-6

[CR10] Kim SH, Choi SM, Kim BC, Choi KH, Nam TS, Kim JT, Lee SH, Park MS, Kim SJ (2017) Risk factors for aseptic meningitis in herpes zoster patients. Ann Dermatol 29:283–287 28566903 10.5021/ad.2017.29.3.283 PMC5438933

[CR11] Kupila L, Vuorinen T, Vainionpää R, Hukkanen V, Marttila RJ, Kotilainen P (2006) Etiology of aseptic meningitis and encephalitis in an adult population. Neurology 66:75–80 16401850 10.1212/01.wnl.0000191407.81333.00

[CR12] Leber AL, Everhart K, Balada-Llasat JM, Cullison J, Daly J, Holt S, Lephart P, Salimnia H, Schreckenberger PC, DesJarlais S, Reed SL, Chapin KC, LeBlanc L, Johnson JK, Soliven NL, Carroll KC, Miller JA, Dien Bard J, Mestas J, Bankowski M, Enomoto T, Hemmert AC, Bourzac KM (2016) Multicenter evaluation of biofire filmarray meningitis/encephalitis panel for detection of bacteria, viruses, and yeast in cerebrospinal fluid specimens. J Clin Microbiol 54:2251–2261 27335149 10.1128/JCM.00730-16 PMC5005480

[CR13] Lee GH, Kim J, Kim HW, Cho JW (2021) Herpes simplex viruses (1 and 2) and varicella-zoster virus infections in an adult population with aseptic meningitis or encephalitis: a nine-year retrospective clinical study. Medicine (Baltimore) 100:e27856 34797322 10.1097/MD.0000000000027856 PMC8601327

[CR14] Mørch K, Fylkesnes SI, Haukenes G (2003) [Meningitis associated with reactivation of varicella-zoster virus]. Tidsskr Nor Laegeforen 123:2871–2873 14600712

[CR15] Nagel MA, Gilden D (2014) Neurological complications of varicella zoster virus reactivation. Curr Opin Neurol 27:356–360 24792344 10.1097/WCO.0000000000000092 PMC4189810

[CR16] Sabbagh B, Seijari MN, Albuni MK, Barakat M, Alfitori GB (2022) Varicella-Zoster meningitis with normal CSF cellularity: a rare case report. IDCases 28:e01484 35392597 10.1016/j.idcr.2022.e01484 PMC8980618

[CR17] Tansarli GS, Chapin KC (2020) Diagnostic test accuracy of the BioFire^®^ FilmArray^®^ meningitis/encephalitis panel: a systematic review and meta-analysis. Clin Microbiol Infect 26:281–290 31760115 10.1016/j.cmi.2019.11.016

[CR18] Troendle M, Pettigrew A (2019) A systematic review of cases of meningitis in the absence of cerebrospinal fluid pleocytosis on lumbar puncture. BMC Infect Dis 19:692 31382892 10.1186/s12879-019-4204-z PMC6683453

[CR19] Trujillo-Gómez J, Tsokani S, Arango-Ferreira C, Atehortúa-Muñoz S, Jimenez-Villegas MJ, Serrano-Tabares C, Veroniki AA, Florez ID (2022) Biofire filmarray meningitis/encephalitis panel for the aetiological diagnosis of central nervous system infections: a systematic review and diagnostic test accuracy meta-analysis. EClinicalMedicine 44:101275 35198914 10.1016/j.eclinm.2022.101275 PMC8851290

[CR20] Tunkel AR, Glaser CA, Bloch KC, Sejvar JJ, Marra CM, Roos KL, Hartman BJ, Kaplan SL, Scheld WM, Whitley RJ (2008) The management of encephalitis: clinical practice guidelines by the infectious diseases society of America. Clin Infect Dis 47:303–327 18582201 10.1086/589747

